# Measuring patient reported outcomes in brachytherapy: Why we should do it and more importantly how

**DOI:** 10.1016/j.ctro.2024.100870

**Published:** 2024-10-02

**Authors:** Aspazia Spyrou, André-Guy Martin, Jean-Michel Hannoun-Lévi, Alexandra Stewart

**Affiliations:** a Royal Surrey Hospital, Guildford, UK; b Centre Intégré de Cancérologie, CHU de Québec – Université Laval, Quebec, Canada; c University of Cote D’Azur, Nice, France; d University of Surrey, Guildford, UK

**Keywords:** Brachytherapy, patient reported outcomes (PROs), Breast cancer, Prostate cancer, Gynaecologic cancer, Rectal cancer

## Abstract

As the treatment for cancer improves and advances are made, the clinical focus is often on treatment response and survival. However, these are not the only factors which are important to patients. More patients are living longer after cancer treatment and therefore it is important that we can describe not only the treatment to patients but also what their life will be like during and after treatment. Patient reported outcomes (PROs) allow us to describe these. Although there are a range of patient reported outcome measures (PROMs) available to the clinician to assess these, the use of them in many areas of brachytherapy lags behind ideal levels. Brachytherapy has many features that differ to external beam radiotherapy (EBRT) yet the assessment of quality of life during and after treatment is much more scarce than EBRT. Brachytherapy is often used in the setting of organ preservation or in place of radical surgery, yet there is a paucity of quality of life data comparing the different treatment modalities. This review article will aim to elaborate on the evidence that exists in the use of specific PROMs within prostate, breast and gynaecologic cancers and describe the development of a novel PROMs approach in rectal brachytherapy which aims to identify and resolve symptoms at an early stage.

## Highlights

• A review of PROs in brachytherapy-especially prostate, gynaecologic and breast cancer.
• To elaborate on the evidence that exists in the use of specific PROMs within prostate, breast and gynaecologic cancers.
• Describing a novel rectal brachytherapy PROMs approach aiming to identify and resolve symptoms at an early stage.

## Search strategy

A pubmed and Google scholar literature search was performed. Due to a large number of results, the search period was narrowed for articles published between 2020 and 2024. The key words used were rectal cancer, cervical cancer, prostate and breast cancer, patient related outcome measures (PROMs), brachytherapy and cancer survivorship. The search words were combined with boolean features such ‘AND’ and ‘OR’, for example ‘cervical cancer and use of PROMs’, or ‘brachytherapy and quality of life’. Studies outside the listed time period were included if they were of particular relevance. The studies that focused on the use of PROMs were used whereas those that evolved around specific interventions and group comparisons were excluded. There were no restrictions on the type of PROMs that were used, the method of information collection and the study design.

## Introduction

With continuing advances in oncology care, the number of cancer survivors is increasing worldwide. While the primary focus is rightly on maximising longevity, the consequences of treatment are often not assessed. Many of the effects of cancer treatment are temporary and will disappear after a few weeks. However, despite efforts to reduce toxicity, patients may still experience long-term effects. Survivors of cancer may continue to experience a range of issues around their cancer therapy;i the late effects associated with it and the emotional distress the entire process brings. Physician assessment of patients’ toxicity often under-reports symptoms and may fail to focus on aspects that are important to patients [1]. Patient-reported outcome measures (PROMs) are the tools and instruments used to report patient reported outcomes (PROs) and assess symptom burden, functional status and psychological or emotional well-being in cancer sufferers and cancer survivors. PROs are defined as *any report of the status of a patient's health condition that comes directly from the patient, without interpretation of the patient's response by a clinician or anyone else* [2]. PROMs are commonly measured using questionnaires, most often delivered on paper, but increasingly through more electronic or interactive methods. Measuring PROMs helps to ensure that the patient’s voice is present in all aspects of their care, ensuring that treatment management remains patient-centred. The questionnaires aim to capture patients’ own opinions and can be used to describe the impact of their individual condition and its treatment on their own life and to take steps to manage their own recovery. Self-management is defined as the individual’s awareness and active participation in their recovery to minimise treatment side-effects and promote survival, health and wellbeing [3].

The National Cancer Survivorship Initiative commenced in the UK in 2008. The initial focus was on improving the diagnosis and treatment of cancer, but it became clear that quality of life data needed to be collected to develop services to enable cancer survivors to live ‘as healthy and as good a quality of life as possible.’ This was used to develop personalised follow-up care pathways for groups of cancer survivors treated with curative intent [4]. Patients were offered a recovery package that focused on self-management including information on general wellbeing, an end of treatment summary and a plan for a review by their general practitioner. Studies demonstrated that 50 % of patients with colorectal cancer, 80 % with breast cancer and 50 % with prostate cancer could safely self-manage and have their needs met. Although this model had the capacity to detect treatment related side effects, the focus was on the risk of recurrence or the development of a new cancer. It became clear that the model needed to be adjusted to account for the presence of co-morbidities, the functional and physical status of the patient, the psychosocial impact of their diagnosis, patients’ literacy and confidence and their ability to self-manage. It was predicted that the incorporation of these dimensions within cancer care in the UK, with further support of the concept of survivorship beyond cancer diagnosis, could not only lead to improvements in the quality of life (QoL) and overall wellbeing but also to savings of around £90 million over 5 years. The perfect model of care incorporates different aspects of cancer management; management of cancer treatment consequences and patient wellbeing, prevention of new cancers and recurrences, surveillance programmes to identify recurrent or new cancer and co-ordination between cancer care specialists and primary general care physicians. One key feature of such a model is the use of PROMs. Effective self-management through PROMs requires evidence-based programmes and tools as well as a proactive model in place [3]. This model must incorporate the collaboration of health care professionals, while ensuring it achieves good education of the patients around symptoms and side effects, an efficient patient follow-up plan, adoption of healthy behaviours by patients and self-management skills by the cancer survivor including problem solving, communicating and goal setting.

PROMs can be the key in achieving supported self-management of a cancer survivor’s symptoms and enhancing overall physical and emotional wellbeing. Promoting the cancer survivor’s capacity and ability to manage their own physical, emotional and psychosocial health can be of critical importance in optimising health, QoL and survivorship experiences. This type of effective self-management, self and disease awareness and own initiative can be supported by such a model whereby there is shared care and communication between the tertiary cancer specialists and the primary general care physicians. This can provide the ultimate support to a cancer patient; with specialist cancer care as needed, by an oncologist or specialist nurse, and the ongoing support from their community health care professional who is more familiar with their long-standing health conditions. In addition, research shows that PROMs may even improve overall survival in cancer patients [5]. However, the implementation of PROMs in cancer care can be complex and time-consuming, sometimes creating significant additional work but without meaningful alterations to cancer care and patient support [6]. There is currently a visible gap as to what are the correct PROMs to be used in various cancer types or treatments and when should they be used.

It is of paramount importance to specifically examine the role of PROMs in patients undergoing brachytherapy as part of their cancer treatment journey. Brachytherapy holds a unique role in radiation therapy. It can deliver highly localised treatments, allowing treatment delivery in greatly decreased time or with the benefits of organ preservation but often with a more procedural approach which may be more intense for the patient to experience. Higher doses per fraction may risk higher toxicity. The avoidance of surgery may deliver organ preservation but, for a patient to make an informed decision, it is important that they have a complete picture of life after treatment, which can be gained with the use of PROMs.

Routine measurement of PROs in brachytherapy varies according to disease site. In prostate brachytherapy, the use of PROMs is commonplace. In other disease sites, routine PROM collection is much less common and reporting of PROs tends to come from clinical trials. Brachytherapy is often combined with external beam radiotherapy (EBRT), making it more challenging to detect individual effects of brachytherapy on long-term QoL. This is where timing the collection of PROMs around individual brachytherapy sessions may be valuable. Strategies to make collection of PROs easier and increase patient adherence are highly desirable. We recognise that PROMs are assessed in a variety of disease sites and it would be impossible to present all of them therefore we present a selection of disease sites where brachytherapy is commonly used and explore how PROMs are used in brachytherapy and describe future developments.

## Gynaecology

Women that have a diagnosis of a gynaecological cancer and are undergoing intensive cancer treatments have a significant symptom burden, including acute and late toxicity from their treatments. There is a long pathway to treatment and recovery and the treatment can leave women with a traumatic memory of their experience, exacerbating the psychological effects they may experience post treatment. Many of these women experience specific challenges such as infertility, menopause, loss of their femininity and impairment of their sexual function while dealing with the more usual toxicities of pelvic radiotherapy such as diarrhoea, urinary frequency and skin breakdown. Many of the psycho-sexual toxicities are not evaluated or discussed in routine appointments, meaning that PROMs are an excellent way to assess these and improve the conversation between a patient and their clinical team.

Andring et al. evaluated the outcomes of women undergoing radiotherapy for gynaecologic cancer, including brachytherapy and also chemotherapy. Questionnaires were used to assess PROs at baseline, after brachytherapy and at 60 days follow-up [7]. The validated questionnaires used were the European Organization for Research and Treatment Cancer (EORTC)-Quality of Life Question (QLQ) Core 30 (C30) [8] and EORTC-QLQ Cervical Cancer Module (CX24) [9]. 29 of the 33 patients enrolled completed the EORTC-QLQ-C30. When the results were compared with a reference population, even at baseline the cervical cancer patients had worse social function scores, fatigue, nausea and vomiting and appetite loss. Although there was no statistical difference in cognitive or role functioning between the two populations, the patients in the study reported a significant psychosocial burden because of their diagnosis with 55 % reporting depression, 66 % expressing interference with their family life and 69 % documenting significant anxiety and worry. When the symptoms were compared at the predefined time points, it was evident that symptoms such as physical function, fatigue and appetite were worse at baseline. At the 60 day follow-up, scores were better with significant improvement of appetite. Although they documented an acute increase in diarrhoea after brachytherapy, this also improved at follow-up, 68 % of patients documented abdominal cramping, 36 % noted urinary leakage and 25 % difficulty controlling their bowels. The patients also reported a significant worsening of their body image perception and femininity. At baseline, 68 % were dissatisfied with their body, 64 % were feeling less attractive and 57 % felt less feminine. Furthermore, 86 % of women said they were not sexually active and 61 % expressed a significant worry about engaging in sexual activity. When compared with the reference populations, it was apparent that women with cervical cancer reported a worsened body image (p < 0.001) and sexual function (p < 0.001). Even though at follow up the patients in this study reported improved symptom burden, there was no improvement in their sexual function, sexual enjoyment, body image or vaginal function. This study demonstrated the importance of a multi-depth approach in how these patients should be consulted. Toxicities such as psychological stress, reduced or challenging social functioning, financial burden are often not obvious in a clinical consultation room. This study underlined the finding that by providing patients with PROMs, previously undetected issues can be captured. The challenge is then to use these findings to improve QoL. A prospective, multi-institutional study aimed to achieve in 80 cervical cancer patients who were attending routine follow-up appointments. All patients were treated with radiotherapy, including brachytherapy, and 78 of them received concurrent cisplatin chemotherapy. The patients were asked to complete the EORTC-QLQ-CX24 questionnaire prior to their clinic visit and review it with their oncologist during the visit. Both patient and oncologist experiences were assessed using a 12-item feedback questionnaire after the clinic visit in 76 patients. Fifty-six percent of patients reported at least one severe symptom and 56 % at least one moderate symptom. The most reported symptoms were ‘have you worried that sex would be painful’ (30 %), ‘do you pass urine frequently’ (47 %), and ‘Have you had hot flushes and/or sweats’ (34 %). The majority of patients felt that the use of a questionnaire greatly improved communications and opened a channel of discussions with their oncologist for those topics that are sensitive and difficult to normally bring up into a clinic consultation.

Kirchheiner et al. investigated the psychological effects of cervical brachytherapy in 50 patients using the Impact of Event Scale-Revision (IES-R) [10] in addition to EORTC-QLQ C30 and CX24 [11] assessing before, during and after treatment and at one week and 3 months of follow up. It was demonstrated that acute stress disorder (ASD) was seen in 30 % of patients one week after treatment and 41 % of patients demonstrated post-traumatic stress disorder (PTSD) at 3 months. The median stress level prior to brachytherapy was 8/10, compared to 3/10 for EBRT and 5/10 for chemotherapy. The patients most at risk of developing PTSD had a poorer performance status, higher depression level or lower emotional functioning on EORTC QLQ C30. A past history of sexual violence also resulted in PTSD post-brachytherapy. Pain was associated with high rates of ASD. Stewart et al. assessed the introduction of inhaled methoxyflurane (IMF) for brachytherapy applicator removal [12]. High anxiety scores of 6/10 were also demonstrated prior to the first insertion of brachytherapy. Prior to the second insertion 36 % of patients remained anxious prior to IMF introduction and 29 % after IMF introduction. Median pain scores on applicator removal dropped significantly from 7/10 to 0/10 after IMF introduction. This demonstrates how PROMs can be used to identify specific time points in the brachytherapy pathway where interventions may be developed that result in improved QoL.

In a systematic review of 27 observational studies using PROMs in endometrial cancer, the most commonly used PROMs were EORTC QLQ C30 (n = 9), short form (36) Health Survey (SF-36 [13] n = 8), the Functional Assessment of Cancer Therapy-General (FACT-G [14] n = 5) and the Female Sexual Function Index (FSFI [15] n = 4) [16]. PROMs were very helpful in demonstrating differences in QoL according to what treatment a patient received. Three studies showed was no significant difference when adjuvant brachytherapy alone was given compared to surgery alone. In addition, 2 of these studies showed no differences in vaginal or sexual function. The addition of EBRT to brachytherapy resulted in significantly poorer QOL compared to brachytherapy alone, though patients who received EBRT plus brachytherapy did not have significantly different QoL to those who had EBRT alone. A review in 2014 of PROs reported in endometrial cancer trials demonstrated marked gaps in PROM collection, prompting a call for a greater focus on this in subsequent endometrial cancer trials [17]. This review also reported that use of brachytherapy alone resulted in a greatly improved QoL compared to EBRT plus brachytherapy with increased late toxicity in the EBRT groups.

## Breast

Brachytherapy as a treatment modality for breast cancer represents a validated irradiation technique in different clinical situations [18]. The timing of catheter implantation, during or after the wide local excision procedure, can impact on patient perception of the brachytherapy and its consequences in terms of QoL [19]. Currently, in breast cancer treatment, there are 3 main indications for the use of brachytherapy: accelerated partial breast irradiation (APBI) for early breast cancers [20], [21], accelerated partial breast re-irradiation for a second breast conservative procedure in the setting of ipsilateral local recurrence [22] and as a boost for high-risk breast cancers [23].

The GEC-ESTRO phase 3 randomized trial demonstrated that 5 and 10-year local relapse rates after APBI with interstitial brachytherapy or whole breast irradiation (WBI) were non significantly different [20], [21]. Physician assessed acute toxicity reported that no grade 4 toxicity was observed while Grade 3 side effects were exclusively seen for early skin toxicity (radiation dermatitis) with 7 % vs. 0.2 % for WBI and APBI respectively (p < 0.0001) [24]. Five-year late toxicity profiles and cosmetic results were similar in patients treated with WBI or APBI but with significantly fewer grade 2–3 late skin side-effects in the brachytherapy group [25]. QoL was fully investigated by using dedicated questionnaires (EORTC QLQ-C30 [8] and breast cancer module QLQ-BR23 [26]) completed before, immediately after irradiation and during follow-up. The PROs reflected the physician reported outcomes, showing that breast symptom scores were significantly higher in the WBI group immediately after radiotherapy. Despite the more invasive nature of treatment delivery, APBI with multicatheter brachytherapy was not associated with worse QoL compared with WBI [27]. The investigators noted this was the largest trial to date to compare QoL in WBI versus APBI.

While APBI with multicatheter brachytherapy used a twice daily schedule for a total irradiation time of 4 to 5 consecutive days, the concept of very APBI (vAPBI) was proposed in phase 2 prospective trials testing APBI performed in 3, 2 or even 1 day (single fraction) [28], [29] showing that the efficacy in terms of local control was comparable to the results obtained after APBI [30]. In a recent GEC-ESTRO study analysing 515 pts treated with vAPBI, grade 2 and 3 late toxicities were observed in 7.2 and 0.6 % respectively (no grade 4) with no difference between 1 and ≥2 treatment days [31]. The SiFEBI phase 2 prospective trial investigated single fraction vAPBI multicatheter brachytherapy in the elderly and specifically analysed QoL and Comprehensive Geriatric Assessment (CGA) [28]. APBI based on a single fraction in older adults with low-risk breast cancer appeared to be feasible with a minimal loss of autonomy regarding Instrumental Activities of Daily Living and no loss of autonomy in Activities of Daily Living, an acceptable decrease in other CGA domains, and with no impact on global QoL [30]. Moving forward, an APBI workflow optimization has been proposed to the patient with a shorter vAPBI procedure based on a single fraction of multicatheter brachytherapy with only one round trip from the patient’s home to the radiation facility [32]. The improvement of QoL for the early breast cancer patient can also be investigated by proposing the omission of endocrine therapy (ET) which is well known to be associated with side effects often leading to treatment discontinuity [33]. In this frame, the EUROPA phase 3 randomised trial is currently recruiting patients with early breast cancer randomising to either APBI alone (EBRT) or ET without irradiation. A single fraction of APBI with multicatheter brachytherapy has also been proposed in this setting [34].

In the setting of a second ipsilateral breast cancer event (2nd iBCE), the standard treatment is salvage mastectomy (SM). However, for low-risk 2nd iBCE, a second breast conservative treatment (2nd BCT) combining lumpectomy plus re-irradiation of the tumour bed (APBrI) provides excellent subsequent local control rates [35]. The GEC-ESTRO published the results of a matched-pair analysis comparing SM vs. 2nd BCT using brachytherapy, showing that no significant difference was observed between the two groups in terms of oncological outcomes [22]. The rates of late grade 3 and 4 toxicities observed after 2nd BCT are about 10 % and less than 1 %, respectively. Cutaneous and subcutaneous fibrosis remain the most frequent late side effects [36]. In this context, QoL improvement is supported by the possibility of avoidance of a mutilating salvage surgery acknowledging that SM even with immediate reconstruction leads to a deep deterioration of body image, self-confidence and QoL [37], [38]. To provide a high level of evidence for 2nd iBCE management between SM and 2nd BCT, the VENUS phase 3 trial will randomise the two salvage treatment options [39]. QoL will be investigated by EORTC QLQ-C30, BR-23 and BR-45 [40] questionnaires in addition to other PROMs and “fear of cancer recurrence” analysis [41].

## Prostate

Routine collection of PROMs is commonplace in all prostate cancer treatment modalities [42]. A range of validated questionnaires are available such as the expanded prostate cancer index composite (EPIC [43]), international prostate symptom scale (IPSS [44]) or the international index of erectile function (IIEF-5 [45]). They provide a patient’s perspectives on the effects of the therapy, adding additional information to the clinician-reported biomedical outcomes. The PROTECT (Prostate Testing for Cancer and Treatment) trial demonstrated that there was no difference in 15 year mortality between active surveillance, radical prostatectomy or radiotherapy in low-risk prostate cancer (brachytherapy was not examined) therefore concluded that treatment decisions should be made by weighing up the trade-offs between benefit and harm [46]. With this approach PROMs become increasingly important. PROs may also give additional information regarding the cost-effectiveness of the selected therapeutic approach [47] and also help to aid patient decision making regarding which treatment modality to choose. Assessment of the IPSS following brachytherapy demonstrated that the urinary toxicity from brachytherapy peaks at 2 weeks following the procedure and returned to baseline at a median of six weeks post procedure [48]. A review of toxicity in the elderly concluded that irradiation using brachytherapy appeared to be well tolerated in terms of QoL and therefore curative management should be not be discouraged in the older age group [49].

A systematic review analysing PROs reported in 60 studies including prostate brachytherapy demonstrated that prostate brachytherapy appears to show lower rates of urinary incontinence and sexual dysfunction compared to radical prostatectomy or EBRT [50]. This may aid decision making if studies demonstrate that treatment modalities appear to be equally efficacious. The SPIRIT trial failed to randomise patients between surgery and brachytherapy but on analysing patients who were either randomised or chose a treatment arm, the patients undergoing brachytherapy demonstrated improved urinary and sexual function and higher patient satisfaction [51]. Combining EBRT and brachytherapy appears to offer a biochemical advantage but at the risk of reports of higher urinary toxicity [52].

Although PRO assessment is commonplace in prostate brachytherapy, the focus is predominantly on urinary toxicity rather than global QoL assessment. In a series of 296 patients with prostate cancer treated with brachytherapy, the effect on long-term QoL was measured using EORTC-QLQ C30 and PR25 [53]. The QLQ-C30 outcomes were compared to an age-stratified German reference population. Interestingly the global quality of life was improved in the brachytherapy groups compared to the general population, pain and financial impact scores were also improved in the brachytherapy cohort. There were minor differences in diarrhoea, constipation and appetite loss but too small to be considered significant. There was not a reference population for the QLQ-PR25 therefore the scores were compared to earlier assessments in the same cohort. There was a trend towards improved urinary symptoms, sexual activity decreased over time and with advancing age but for those that remained sexually active over the two time points, the sexual function remained the same.

When brachytherapy is used for reirradiation for prostate cancer, it appears to show lower patient reported toxicity when compared to other techniques, salvage radical prostatectomy, high intensity focussed ultrasound or cryotherapy, particularly for erectile dysfunction [54]. In contrast a review of focal therapies including eight studies of focal brachytherapy, showed that erectile function decreased after treatment in 3 studies, in one study by as much as 50 % [55].

## Rectal

Brachytherapy in rectal cancer is increasing in uptake, particularly after the OPERA trial demonstrated improved local control with the use of a contact brachytherapy boost over chemoradiotherapy alone when aiming to achieve organ preservation for early rectal cancer [56]. However, the routine assessment of PROs has lagged behind other areas such as prostate brachytherapy. The National Institute of Health and Care Excellence (NICE) in the UK published ‘Improving outcomes in colorectal cancer’; guidance on cancer services providing key recommendations for the improvement of health outcomes in colorectal cancer. This guidance makes recommendations for future research, stating that, since the treatment of colorectal cancer leads to very specific side effects relating to bowel function, colorectal cancer specific PROMs should be developed to standardise the interpretation of quality-of-life measures as secondary endpoints for future clinical trials. NICE stated that better information generated via PROMs could potentially enhance quality of care, inform commissioning and promote patient choice. This is of pivotal importance especially within the context of an organ preservation approach. Organ preservation options allow patients with complete or near-complete pathological responses after radiotherapy with or without concurrent chemotherapy, to avoid radical surgery, the significant co-morbidities associated with it, all while preserving anorectal function and therefore potentially maintaining a better QoL.

However, within the context of the non-operative management of rectal cancer, there is lack of data for PRO assessment regarding the optimal time points for assessments, appropriate follow-up schedules, selected anorectal function tests if required and QoL assessment tools. The international consensus recommendations for key outcome measures for organ preservation after radiotherapy for rectal cancer agreed that the EORTC QLQ-C30 is the standard method of QoL assessment and should always be used [57]. The consensus group defined a list of the ten most important symptomatic toxicity items to be evaluated as part of a patient reported assessment; bowel urgency, faecal incontinence, bowel frequency, diarrhoea, tenesmus, toilet dependency, night-time bowel opening, urinary urgency, impotence and pain. There was some agreement (42 %) that the EORTC QLQ-CR29 should be used in addition to QLQ-C30, however they recognised that although the CR29 questionnaire covers many aspects of bowel, urinary, stoma and sexual function symptoms but it does not include all the symptoms that could occur following a non-surgical approach, specifically symptoms such as bowel urgency and toilet dependency. These are included in the low anterior resection syndrome (LARS) score although it fails to ask questions on urinary and sexual function. The group recommended that toxicity assessments should be documented at baseline, 3 months, 12 months, 24 months, 36 months and 60 months after treatment.

CITRuS1 (NCT04697394) is a UK multi-centre study aiming at investigating the feasibility of answering PROMs using electronic means, assessing multiple symptoms and side effects in a longitudinal fashion, aiming to recruit 380 patients over 2.5 years. The study uses standard QoL questionnaires such as EORTC QLQ-CR29 and QLQ-C30 but also includes more holistic assessments such as the Pittsburgh Sleep Quality Index (PSQI) and the PASE, Patients will complete all questionnaires at baseline (excluding decision regret which can only be assessed after treatment has occurred) and then selected questionnaires each month. The patients complete baseline assessments, followed by a selection of questionnaires every month for 3 months, repeating for a total of 2 years of follow up. The trial is designed as a fully electronic pathway with minimal researcher input partly to minimise costs but also to modernise the data collection approach and enable more rapid reporting of toxicity. Electronic data can allow the use of artificial intelligence to detect symptoms and symptom clusters at an earlier stage. This may allow earlier delivery of interventions to improve QoL. The study includes patients receiving surgery as well as those who chose an organ preservation approach using brachytherapy, allowing comparison of QoL between groups. This is important because it is frequently assumed that formation of a stoma results in a poorer QoL that an organ preservation approach but direct comparison using PROMs has not been reported.

CITRuS2 will be a pilot study aiming to use the electronic questionnaires used in CITRuS1 to detect toxicity and to deliver electronic interventions targeting defined symptoms as experienced by various patient cohorts. The delivery of the interventions will be assessed in addition to the acceptability of them and adherence to any recommendations. CITRuS3 will be a larger scale randomised control study, investigating the automatic delivery of interventions compared to routine PROMs collection, assessing the cost-effectiveness of PROMs with automatic interventions in rectal cancer care during and post treatment. The CITRuS study is a unique trial investigating multiple cohorts of colorectal cancer patients simultaneously as they get assigned to different treatment modalities between radical surgeries, chemoradiotherapy options and organ preservation approach with brachytherapy. As in other areas of medicine, the use of computers for the development of electronic PROMs facilitates communication between physicians and patients [58]. Adopting ePROMs will help shape the future of cancer care, improve health equity and contribute to a greener world [59], therefore development of tools to measure these quickly and easily in a cost effective manner that is acceptable to patients are highly desirable. It is important that electronic PROMs do not disadvantage certain groups, such as the elderly or those without access to technology, therefore they must be developed in a way that assesses feasibility and acceptability and ensures delivery to as wide a range of users as possible.

## Conclusions

The reporting of PROs has a crucial role in guiding oncologists and patients in management of disease-related symptoms and treatment-related side effects. By ensuring the use of PROMs, delivered at appropriate time points throughout and after treatment, symptoms can be addressed and interventions delivered to improve quality of care and QoL. Selecting a PROM or a set of PROMs for a specific cancer survivor population based on tumour type, patient demographics, treatment options and treatments completed could be a breakthrough in PROMs implementation and cancer survivorship [60]. We have demonstrated that specific and evidence-based PROMs can be incorporated in the care of brachytherapy patients with prostate, breast, gynaecological and colorectal cancers. The development of PROMs which are specifically aimed at brachytherapy patients would be extremely valuable and aid patient and clinician choice in the future. Use of PROMs, particularly those delivered electronically, can therefore lead to the design of specific interventions aiming at managing symptoms and side effects allowing a new era of consistent, evidence-based, cost-effective cancer care and cancer survivorship.

An important highlight of PROMs is that self-monitoring, [3] and reporting by the patients themselves are essential elements in achieving good usage and success, and therefore to optimise their usefulness and impact, the timing of when to be used within specific patient cohorts needs to be well understood. Furthermore, it is crucial to emphasize that electronic PROMs appear to the most efficient way of delivering PROMs. The most effective PROMs should provide immediate scoring with real time feedback to patient and the health care professional involved. As brachytherapy trials are developed, measurement and reporting of PROMs should be considered an essential element. Future development of mechanisms to deliver PROMs to all patients, whether in trials or not, in a simple, cost-effective way should be a clinical priority which is likely to deliver great benefit to everyone, patients and clinical staff alike.

## Declaration of competing interest

The authors declare that they have no known competing financial interests or personal relationships that could have appeared to influence the work reported in this paper.
